# Clinical Application of Patient-Specific Bolus Based on Molding and Casting Method in Radiotherapy

**DOI:** 10.3390/jcm14113796

**Published:** 2025-05-28

**Authors:** Jaeman Son, Seonghee Kang, Jegal Jin, Hyojun Park, Inbum Lee, Yoonsuk Huh, Chang Heon Choi, Jung-in Kim, Hong-Gyun Wu

**Affiliations:** 1 Department of Radiation Oncology, Seoul National University Hospital, Seoul 03080, Republic of Korea; jaeman0410@snuh.org (J.S.); kangsh012@snuh.org (S.K.); 5d913@snuh.org (J.J.); 5d970@snuh.org (I.L.); 5d969@snuh.org (Y.H.); dm140@snu.ac.kr (C.H.C.); 2 Department of Radiation Oncology, Seoul National University College of Medicine, Seoul 03080, Republic of Korea; 3 Institute of Radiation Medicine, Seoul National University Medical Research Center, Seoul 03080, Republic of Korea; 4 Department of Radiation Oncology, Chung-Ang University Gwang Myeong Hospital, Gwang Myeong 14353, Republic of Korea; phj0318@cauhs.or.kr

**Keywords:** patient-specific bolus, molding and casting, clinical use

## Abstract

**Background/Objectives**: The use of a patient-specific bolus in radiation therapy is critical for achieving precise dose delivery, particularly for irregular anatomical surfaces. Conventional boluses often suffer from poor conformity and air gaps, leading to suboptimal dose distribution. This study aimed to develop and evaluate a novel bolus fabrication method using the mold-and-casting (M&C) technique, which integrates 3D printing and flexible silicone materials to address these limitations. **Methods**: The proposed workflow includes CT imaging, 3D modeling, mold fabrication via 3D printing, and silicone casting to produce a patient-specific bolus. The process is followed by quality assurance steps and clinical application. Geometric accuracy was assessed through surface matching and cross-sectional comparisons, and dosimetric performance was evaluated using in vivo measurements with MOSFET detectors. The biocompatibility of the silicone material was tested according to standardized cytotoxicity, skin sensitization, and irritation protocols. **Results**: The fabricated boluses demonstrated high geometric fidelity, with volumetric and surface discrepancies of less than 3% compared to the planned structures. Dosimetric evaluations indicated that maximum dose differences remained within the clinically acceptable range of ±5%, confirming accurate dose delivery. Biocompatibility tests confirmed that the silicone material is safe for clinical use. **Conclusions**: The M&C method offers a streamlined approach to patient-specific bolus fabrication that integrates well into existing clinical workflows. Compared to traditional sheet boluses, it significantly reduces air gaps and enhances surface dose uniformity. These findings support the clinical potential of this technique to improve both precision and efficiency in radiation therapy.

## 1. Introduction

High-energy megavoltage (MV) photon beams are a cornerstone of external radiation therapy for tumor treatment due to their exceptional tissue-penetrating capabilities. These beams exhibit a build-up region where the maximum dose is delivered at a specific depth beneath the surface, a phenomenon resulting from the uneven distribution of secondary electrons emitted by high-energy photons [1,2,3]. This characteristic creates a skin-sparing effect, which reduces the dose to the surface tissues and mitigates skin-related complications. This advantage is particularly significant for the treatment of deep-seated tumors. However, this very feature presents a challenge when treating superficial tumors located near the skin surface, as it limits the dose delivered to the tumor.

To address this limitation, a bolus is commonly placed directly on the patient’s skin to enhance surface dose delivery. The bolus also plays a critical role in achieving dose uniformity by compensating for irregularities in skin or tissue contours [4,5,6,7,8,9]. While commercially available sheet boluses, typically made from soft, rubbery tissue-equivalent materials, are widely used, they frequently result in undesired air gaps between the bolus and the skin, particularly over irregular contours. Such air gaps, as reported by Butson et al., can range from 4 to 10 mm and cause dose reductions of up to 10%, especially under 6-MV photon beams [10].

To mitigate these challenges, researchers have explored various alternatives, including the use of paraffin wax and advancements in 3D printing technology [11,12,13,14,15]. Customized boluses made from paraffin wax have demonstrated reduced complications compared to standard sheet boluses, but issues related to achieving consistent thickness remain a concern. Meanwhile, the emergence of 3D printing technology has enabled the direct fabrication of personalized boluses tailored to individual patient anatomy. Despite offering improved dose distribution, the rigidity of 3D-printed materials presents limitations in cases requiring flexibility to conform closely to irregular surfaces [16,17,18,19].

To address these limitations, researchers have recently introduced a mold and casting (M&C) method integrated with 3D printing. Among the materials tested for M&C bolus fabrication, Dragon Skin™ 10 MEDIUM, a platinum-cure liquid silicone, has shown significant promise [20]. This material’s low shore hardness provides greater flexibility than other alternatives, reducing air gap formation and shortening production times while exhibiting favorable biological characteristics. However, Dragon Skin’s high viscosity complicates the injection process, requiring extended curing times and prolonging the overall fabrication workflow [21,22,23].

Building upon these advancements, we developed a patient-specific bolus with lower shore hardness and reduced curing time, which improves flexibility, minimizes air gaps, and streamlines clinical workflow. Its improved flexibility ensures a closer fit to irregular anatomical contours, minimizing air gaps and optimizing dose delivery. In contrast, the bolus developed in this study utilizes a silicone material with lower shore hardness, improving its adaptability to complex anatomical surfaces while maintaining structural integrity.

One major challenge in bolus application is preventing unwanted air gaps, as conventional 3D-printed boluses often struggle to conform due to material rigidity. The M&C method effectively addresses this issue by casting the bolus in a precisely molded form, ensuring optimal skin contact and minimizing air gaps. The M&C method efficiently utilizes 3D printing for mold creation, reducing preparation time and complexity for clinical use. This approach significantly reduces preparation time, curing duration, and overall complexity, making it more suitable for clinical implementation. The material’s biocompatibility was confirmed through standard tests. Though Dragon Skin™ has demonstrated good biocompatibility, its higher viscosity and prolonged curing process present additional handling challenges. As a result, the M&C-based patient-specific bolus developed in this study offers greater flexibility, improved skin conformity, reduced air gaps, enhanced clinical workflow efficiency, and validated biocompatibility.

In this study, we investigate the physical, biological, and dosimetric properties of this newly developed patient-specific bolus. The dosimetric properties were evaluated across various anatomical sites in clinical settings and validated through in vivo dosimetry, demonstrating its potential for enhancing the precision and efficacy of radiation therapy.

## 2. Materials and Methods

### 2.1. Patients and Treatments

Before this research started, we obtained IRB (Institutional Review Board) approval for evaluating the clinical application of the patient-specific bolus. This study was conducted on six patients who were treated at Seoul National University Hospital and consented to participate in the clinical trial, with detailed information regarding each patient’s treatment site presented in Table 1.

### 2.2. Clinical Workflow for the Fabrication of Patient-Specific Bolus

**Figure 1:**
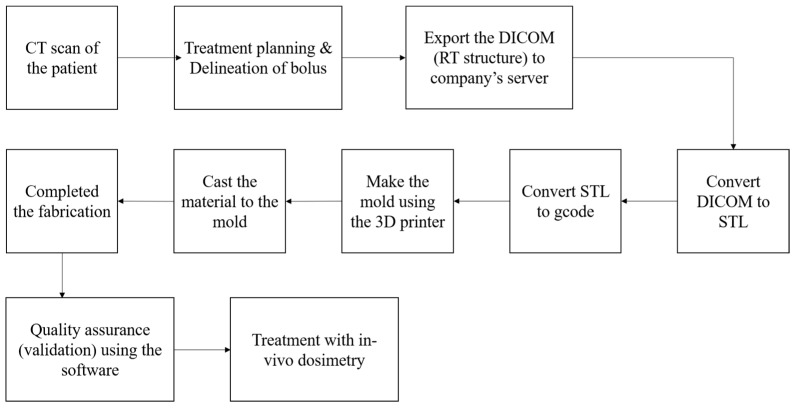
Workflow for fabricating a patient-specific bolus using the M&C method.

To utilize the patient-specific bolus, the clinical workflow illustrated in Figure 1 is followed for manufacturing and treatment. Initially, the patient undergoes planning CT to prepare for the production of the patient-specific bolus. A bolus contour is then delineated on the region requiring bolus application. The RT structure file containing the contour information is exported from the TPS. The RT structure is converted into an STL file, which is further processed into a gcode file compatible with 3D printing. We utilized the M300 Dual 3D printer (Zortrax, Olsztyn, Poland) for mold fabrication, using Z-HIPS as the printing material in this study. Z-HIPS was selected due to its high impact resistance, making it well-suited for mechanical prototyping and performance testing. Additionally, ease of separation from the silicone was a critical consideration. To facilitate smooth demolding after curing, a release agent was applied to the inner surface of the mold prior to casting the silicone. Using the fabricated mold, the bolus material (silicone, Shenzhen Inibaba Technology Co., Ltd., Shenzhen, China) is cast and allowed to cure. After a specified curing period, the mold is removed, and the bolus is finalized. The completed bolus is delivered back to the hospital, where quality assurance is performed via CT imaging. Geometric accuracy is evaluated using Polyworks 2014 software (InnovMetric Software Inc., Québec City, QC, Canada). If the geometric accuracy meets the specified criteria, the fabricated bolus is applied for treatment.

### 2.3. Physical & Biological Evaluation

The patient-specific bolus is composed of opaque white silicone rubber. The physical properties of the bolus were verified by testing facilities (Huizhou Hongyejie Technology Co., Ltd., Shenzhen, China, and Koptri, Seoul, Republic of Korea).

To ensure the product’s safe use for patients, a series of biological stability assessments were conducted, including tests for cytotoxicity, skin sensitization, and skin irritation. Cytotoxicity was analyzed using L-929 mouse fibroblast cells through the Elution method, adhering to ISO 10993-5:2009 “In Vitro Cytotoxicity Tests” and ISO 10993-12:2021 (E) guidelines [24,25]. Skin sensitization and irritation studies were carried out on guinea pigs and New Zealand rabbits, respectively, following ISO standards 10993-10:2021 (E) “Tests for Skin Sensitization”, ISO 10993-23:2021 (E) “Tests for Irritation”, and ISO 10993-12:2021 (E) “Guidelines for Sample Preparation and Reference Materials” [26,27]. The experiments were performed at BIONEEDS India Pvt. Ltd., Karnataka, India, an institution certified for Good Laboratory Practice and accredited by the Association for Assessment and Accreditation of Laboratory Animal Care.

### 2.4. Evaluation of Geometric Accuracy

To assess geometrical accuracy, the shape of the fabricated bolus was compared to the planned bolus structure generated in the TPS [28]. CT imaging of the fabricated bolus was performed under the same conditions used for the patient. The resulting bolus image was then converted into an STL file to facilitate comparison using mesh data. For surface matching, registration was performed with Polyworks 2014 software. This software applies a cloud-based surface matching technique based on the iterative closest point (ICP) algorithm. The ICP algorithm minimizes the distance between a point in one cloud set and its nearest neighbor in the other set by iteratively adjusting transformation parameters. Geometry differences were analyzed by measuring the average distance between points, and a cross-sectional profile of the aligned bolus geometry was examined. Additionally, basic geometric parameters such as surface area and volume were calculated for further evaluation.

### 2.5. Dosimetric Evaluation

We performed in vivo dosimetry for dose evaluation [29]. In vivo dosimetry measurements were conducted using 3~5 MOSFET detectors (TN-502RD-H, Best Medical Canada Ltd., Ottawa, ON, Canada), depending on the size of the bolus, with the detectors positioned between the bolus and the patient’s skin in PTV volume. The MOSFET we used has an active region of 0.2 × 0.2 mm^2^, which is extremely small, and the SiO_2_ thickness is only 0.5 μm. Prior to beam delivery, all linear accelerators were calibrated for output following the American Association of Physicists in Medicine Task Group 51 protocol, ensuring consistency and accurate dose delivery. The dose measured by the MOSFET detectors was compared to the dose calculated by the TPS at the same points of interest. This comparison was used to evaluate the accuracy of the delivered dose and to identify any discrepancies with the planned dose. To ensure the reliability of the measurements, the MOSFET detectors were pre-calibrated under clinical conditions prior to their use.

## 3. Results

### 3.1. Physical & Biological Evaluation

The physical properties of patient-specific bolus are detailed in Table 2. These characteristics underscore its suitability for achieving precise fitting to irregular anatomical surfaces, thereby reducing the formation of air gaps during clinical use.

The biological compatibility of patient-specific bolus was assessed through a series of standardized tests, including cytotoxicity, skin irritation, and skin sensitization evaluations. Cytotoxicity testing was conducted using L-929 mouse fibroblast cells, where less than 20% of the cells exhibited morphological changes. The material caused minimal growth inhibition and demonstrated grade 1 reactivity on the cytotoxicity scale, with grade 0 indicating no reactivity (control) and grade 4 representing complete destruction of cell layers. Skin irritation testing was performed on New Zealand white rabbits, with no signs of erythema or edema observed up to 72 h after the removal of the test patches. Similarly, skin sensitization testing conducted on guinea pigs revealed no visible reactions within 48 h following the application of patient-specific bolus extract or the removal of patches. These findings confirm that patient-specific bolus is non-cytotoxic, non-irritating, and non-sensitizing. The results validate its safety and demonstrate its suitability for clinical applications in patient treatments.

### 3.2. Evaluation of Geometric Accuracy

**Figure 2:**
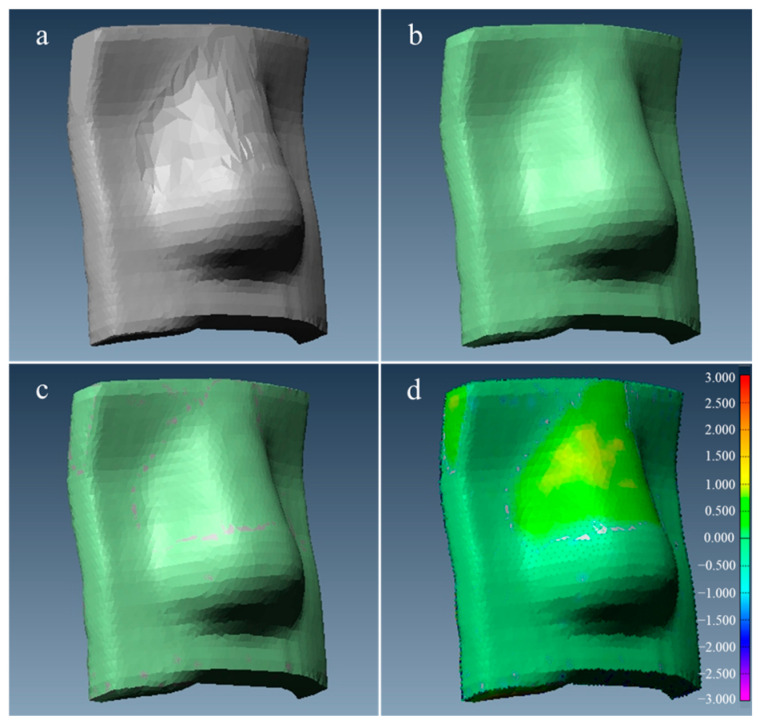
Comparison of the planned and fabricated bolus structures: (**a**) bolus structure as planned in the TPS; (**b**) fabricated bolus structure as measured; (**c**) overlaid structures following registration; (**d**) color map showing the distance differences between the two structures (units: mm).

The geometric accuracy of the fabricated bolus was assessed by comparing its structure to the planned bolus generated in the treatment planning system (TPS). Figure 2 illustrates the process: (a) the bolus structure as planned in the TPS; (b) the fabricated bolus structure measured via CT; (c) the overlaid structures after registration; (d) a color map indicating the distance differences between the planned and fabricated structures. These comparisons provide a visual confirmation of the alignment between the two structures.

**Figure 3:**
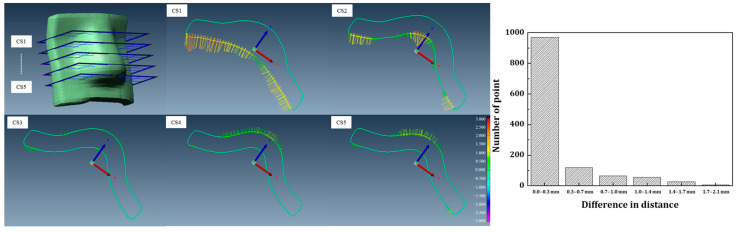
Histogram of surface distance differences between the planned and fabricated bolus (units: mm).

Figure 3 presents an evaluation of the geometric accuracy of the fabricated bolus by overlaying the planned and fabricated bolus structures and analyzing five cross-sectional profiles. A histogram is provided to illustrate the distribution of surface distance differences. A total of 1245 measurement points was analyzed, of which 1156 points (92.8%) exhibited deviations within 1 mm, demonstrating a high level of geometric fidelity. The data presented in this figure further support the precision and reliability of the patient-specific bolus fabrication method.

Quantitative analysis was conducted using the cross-sectional profiles of the registered structures for each patient, as presented in Table 3. The average distance differences between the planned and fabricated bolus cross-sections ranged from (0.1 ± 0.1) mm to (0.5 ± 0.6) mm in patient 6. The variability was within acceptable limits, confirming the reliability of the mold and casting (M&C) method in achieving accurate geometrical reproduction of patient-specific bolus designs.

In addition to cross-sectional comparisons, Table 4 presents the differences in volume and surface area between the planned and fabricated boluses. The percentage differences in volume ranged from 0.9% to 3.0%, while the differences in surface area ranged from 0.7% to 2.9%. These minimal discrepancies further demonstrate the precision of the fabrication process.

Overall, the geometric accuracy evaluation indicates that the M&C method can produce boluses with high fidelity to the planned designs, ensuring consistent coverage of the target treatment area and minimal air gaps. This precision is critical for optimizing dose delivery and enhancing the efficacy of radiation therapy.

### 3.3. Dosimetric Evaluation

**Figure 4:**
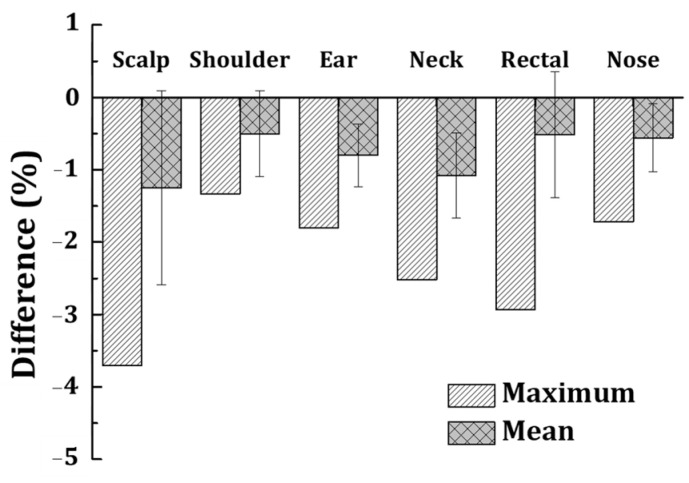
In vivo dosimetry results for patient-specific bolus validation.

The dosimetric evaluation focused on validating the dose delivery accuracy of the patient-specific bolus through in vivo dosimetry. The in vivo dosimetry results obtained using MOSFET detectors demonstrated that the mean dose differences between the measured and planned values were within clinically acceptable limits across all treatment sites. The calculated mean dose differences, along with their standard deviations, were as follows: scalp: (1.25% ± 1.34%), shoulder: (0.50% ± 0.59%), ear: (0.80% ± 0.43%), neck: (1.08% ± 0.59%), rectal: (0.52% ± 0.87%), and nose: (0.56% ± 0.47%). Furthermore, Figure 4 confirms that the maximum dose differences remained within the clinically acceptable range of ±5%, validating the accuracy and reliability of the M&C-based patient-specific bolus for radiation therapy applications. These results indicate that the patient-specific bolus not only compensates for tissue irregularities but also minimizes the impact of air gaps, which are often a concern with conventional bolus materials. The bolus’s superior fit and flexibility contributed to its ability to maintain consistent contact with the patient’s surface, leading to precise dose delivery. Additionally, the statistical analysis of the dosimetric data revealed that the variability among patients was negligible, further validating the reproducibility of the bolus’s performance. These findings highlight the effectiveness of the patient-specific bolus in clinical settings, especially for treatments requiring precise surface dose modifications. The results also suggest that the bolus fabrication process and material properties play a crucial role in achieving such accuracy. This evaluation supports the integration of patient-specific bolus into routine clinical workflows, demonstrating its potential to enhance treatment quality while reducing the risks associated with dose delivery inaccuracies.

## 4. Discussion

One of the primary advantages of the proposed patient-specific bolus is its minimal impact on the existing workflow in radiation therapy. The M&C method we employed does not require iterative processes or extended preparation times, allowing for clinical implementation without modifications to the existing workflow. This process seamlessly integrates into the clinical workflow, from CT imaging and contour generation in the treatment planning system (TPS) to bolus fabrication and quality assurance. This efficiency reduces time and labor requirements while maintaining high geometric and dosimetric accuracy. The dosimetric evaluation demonstrated the reliability of the fabricated bolus in achieving accurate dose delivery. As shown in Figure 4, the maximum dose differences measured with MOSFET detectors were consistently within the clinically acceptable range of ±5% for all patients [30]. This result highlights the effectiveness of the bolus in compensating for tissue irregularities and minimizing air gaps, which are common issues with commercial boluses. Furthermore, the statistical analysis showed negligible variability in dose discrepancies among patients, validating the reproducibility of the fabrication process and the performance of the bolus material. The geometric accuracy achieved in this study is critical for ensuring precise dose coverage. The comparison between planned and fabricated boluses revealed minimal discrepancies in volume and surface area, as detailed in Table 3 and Table 4. These small differences reduce the likelihood of dose variations caused by air gaps and ensure uniform dose distribution, which is particularly important for treating superficial tumors. The accurate reproduction of the bolus geometry further demonstrates the suitability of the M&C method for clinical use. The observed distances of 1–3 mm between the fabricated bolus and the planned structures fall within the expected accuracy range of the mold-and-casting (M&C) method. Geometric accuracy analysis showed that the average distance differences between the planned and fabricated boluses ranged from (0.1 ± 0.1) mm to (0.5 ± 0.6) mm. The discrepancies in volume and surface area were within 0.9% to 3.0% and 0.7% to 2.9%, respectively, demonstrating high fidelity in reproduction. Given the production method, the expected precision between the planned and fabricated bolus was within ±2 mm in most cases, which aligns with the inherent variability of the M&C fabrication process. The use of low-hardness silicone (36 Shore 00) ensures flexibility and close adherence to complex anatomical surfaces, effectively compensating for minor geometric deviations and minimizing potential air gaps. These characteristics contribute to the reliability of the patient-specific bolus in clinical applications, ensuring accurate dose delivery without significant impact from minor variations in bolus structure.

The material properties of the bolus contribute significantly to patient comfort and treatment efficacy. The low shore hardness and high elongation of the silicone material allow it to conform closely to irregular anatomical surfaces, ensuring consistent contact with the skin and reducing the potential for pressure points. Biological evaluations confirmed that the material is non-cytotoxic, non-irritating, and non-sensitizing, further supporting its clinical applicability. These characteristics make the bolus a viable solution for various treatment sites, including anatomically challenging regions such as the scalp, shoulder, ear, neck, rectum, and nose. The consistent performance observed across these diverse anatomical sites highlights the versatility of the bolus in adapting to different clinical scenarios. While the current M&C fabrication process demonstrates high accuracy and efficiency, there is potential for further optimization. Advancements in 3D printing technology could reduce the curing time and enhance the precision of mold production. Additionally, integrating automated quality assurance tools could streamline the geometric accuracy evaluation process. These improvements would further enhance the clinical utility of patient-specific boluses. Compared to conventional sheet boluses, the patient-specific bolus developed in this study addresses significant limitations such as air gap formation and inadequate dose delivery over irregular surfaces. The M&C method also offers a cost-effective alternative to fully 3D-printed boluses, balancing precision with practicality. This makes it a promising option for routine clinical implementation.

In future studies, we plan to explore methods for mold fabrication without using 3D printing. While 3D printing has the advantage of accurately replicating the patient’s surface information, it has the significant drawback of being time-consuming. In particular, for keloid treatment, radiation therapy must begin within 72 h after surgery, making it essential to minimize processing time as much as possible [31]. Although we are currently optimizing operations to meet treatment schedules, reducing 3D printing time would allow for a more flexible and efficient patient treatment process. One of the key approaches we are considering is utilizing CNC (Computer Numerical Control) technology to replicate the patient’s surface information. Additionally, we are continuously researching suitable mold materials to enhance this approach. These future improvements aim to further streamline the fabrication process and increase the clinical applicability of patient-specific boluses in radiation therapy.

## 5. Conclusions

In conclusion, this study demonstrates the feasibility and effectiveness of patient-specific boluses fabricated using the M&C method. The boluses achieve high geometric and dosimetric accuracy, integrate seamlessly into clinical workflows, and offer significant advantages over conventional approaches. Further research should focus on refining the fabrication process and exploring the application of this technology in other areas of radiation therapy, such as particle beam treatments, to enhance the precision, efficiency, and accessibility of personalized radiation therapy solutions.

## Figures and Tables

**Table 1:** Information about treatment sites and volume of PTV in TPS.

Patient	Treatment Site	Volume of PTV in TPS
Patient 1	Scalp	1076.2 cm^3^
Patient 2	Shoulder	150.2 cm^3^
Patient 3	Ear	36.0 cm^3^
Patient 4	Neck	5.7 cm^3^
Patient 5	Rectal	716.5 cm^3^
Patient 6	Nose	93.8 cm^3^

**Table 2:** Physical properties of material of patient-specific bolus.

Property	Index
Viscosity (cps)	14,000 ± 1000
Shore Hardness	36 Shore 00
Tear strength (kN/m)	7.5 + 2.0/−2.0
Tensile strength (Mpa)	2.1 + 1.0/−1.0
Shrinkage (%)	≤0.1
Elongation (%)	900~1000
Density (g/cm)	1.02

**Table 3:** Geometric difference of cross-section profile for each patient.

	CS 1 (mm)		CS 2 (mm)		CS 3 (mm)		CS 4 (mm)		CS 5 (mm)	
	Max.	Ave.	Max.	Ave.	Max.	Ave.	Max.	Ave.	Max.	Ave.
Patient 1	2.6	0.6 ± 1.6	2.1	0.8 ± 1.9	2.2	0.7 ± 1.8	2.4	0.8 ± 2.0	2.1	0.7 ± 1.7
Patient 2	1.6	0.4 ± 1.0	2.2	−0.4 ± 1.4	2.1	0.9 ± 1.4	1.9	−0.4 ± 1.1	2.2	−0.4 ± 1.3
Patient 3	2.3	0.7 ± 1.5	2.1	−0.6 ± 1.2	1.9	−0.4 ± 1.3	2.4	−0.7 ± 1.6	2.4	−0.3 ± 1.5
Patient 4	1.9	0.6 ± 1.3	2.2	0.7 ± 1.4	2.1	−0.8 ± 1.3	2.2	0.9 ± 1.5	2.8	0.6 ± 1.9
Patient 5	2.1	0.6 ± 1.6	2.3	0.8 ± 1.4	2.6	0.7 ± 1.9	1.9	0.9 ± 1.1	2.4	0.9 ± 1.6
Patient 6	1.9	0.5 ± 0.6	1.6	0.3 ± 0.4	0.6	0.1 ± 0.4	0.6	0.1 ± 0.1	0.6	0.1 ± 0.3

**Table 4:** Comparison of volume and surface between planned and fabricated bolus.

	Volume			Surface		
	Planned (cm^3^)	Fabricated (cm^3^)	%Diff.	Planned (cm^2^)	Fabricated (cm^2^)	%Diff.
Patient 1	1044.6	1016.0	2.7	2788.1	2719.0	2.5
Patient 2	148.7	144.3	3.0	377.6	367.1	2.8
Patient 3	367.8	372.0	1.1	419.0	416.2	0.7
Patient 4	27.3	27.8	1.8	135.2	132.1	2.3
Patient 5	314.2	305.5	2.8	695.5	675.4	2.9
Patient 6	32.2	31.9	0.9	153.2	149.8	2.2

## Data Availability

The original contributions presented in the study are included in the article, further inquiries can be directed to the corresponding author.

## Abbreviations

The following abbreviations are used in this manuscript:

MV	megavoltage
M&C	mold and casting
IRB	Institutional Review Board
TPS	treatment planning system
ICP	iterative closest point
